# Subcellular Localization of Carotenoid Biosynthesis in *Synechocystis* sp. PCC 6803

**DOI:** 10.1371/journal.pone.0130904

**Published:** 2015-06-17

**Authors:** Lifang Zhang, Tiago Toscano Selão, Eva Selstam, Birgitta Norling

**Affiliations:** 1 School of Biological Sciences, Nanyang Technological University, Singapore; 2 Department of Plant Physiology, Umeå Plant Science Center, University of Umeå, Umeå, Sweden; CEA-Saclay, FRANCE

## Abstract

The biosynthesis pathway of carotenoids in cyanobacteria is partly described. However, the subcellular localization of individual steps is so far unknown. Carotenoid analysis of different membrane subfractions in *Synechocystis* sp. PCC6803 shows that “light” plasma membranes have a high carotenoid/protein ratio, when compared to “heavier” plasma membranes or thylakoids. The localization of CrtQ and CrtO, two well-defined carotenoid synthesis pathway enzymes in *Synechocystis*, was studied by epitope tagging and western blots. Both enzymes are locally more abundant in plasma membranes than in thylakoids, implying that the plasma membrane has higher synthesis rates of β-carotene precursor molecules and echinenone.

## Introduction

Carotenoids are widely found in all oxygenic photoautotrophic organisms, generally embedded in their cellular membranes. Due to their chemical and physical properties, carotenoids influence diverse membrane properties, including fluidity and polarity [1]. They have important roles in protecting organisms against photo-oxidation and photo-inhibition [2–4]. This role is correlated with their ability to quench excited chlorophyll a (Chl a) triplet states which can generate toxic singlet oxygen [5]. Carotenoids are also believed to be essential in the synthesis, accumulation and maintenance of the integrity of the photosynthetic apparatus [6].

Cyanobacteria are a large and diverse group of oxygenic, photosynthetic bacteria. Different species and strains of cyanobacteria have different carotenoid compositions [7–9]; growth conditions, such as growth stage, light intensity [10,11], nitrogen source [12] and even the strain type within a given species can affect the types and amount of carotenoids as well. The major carotenoids in cyanobacteria are β-carotene, its hydroxyl derivatives, zeaxanthin and nostoxanthin, its keto derivatives, echinenone and canthaxanthin and the carotenoid glycosides, myxol-2’-glycosides and oscillol-2,2’-diglycosides. *Synechocystis* sp. PCC 6803 is a widely used model organism in many research areas, such as membrane organization, stress responses, metabolic pathways and recently also in synthetic biology studies. In *Synechocystis*, the major carotenoid components are β-carotene (26%), myxoxanthophyll (36%), zeaxanthin (14%) and echinenone (18%), with other minor carotenoids comprising the remaining 6% [7].

**Fig 1:**
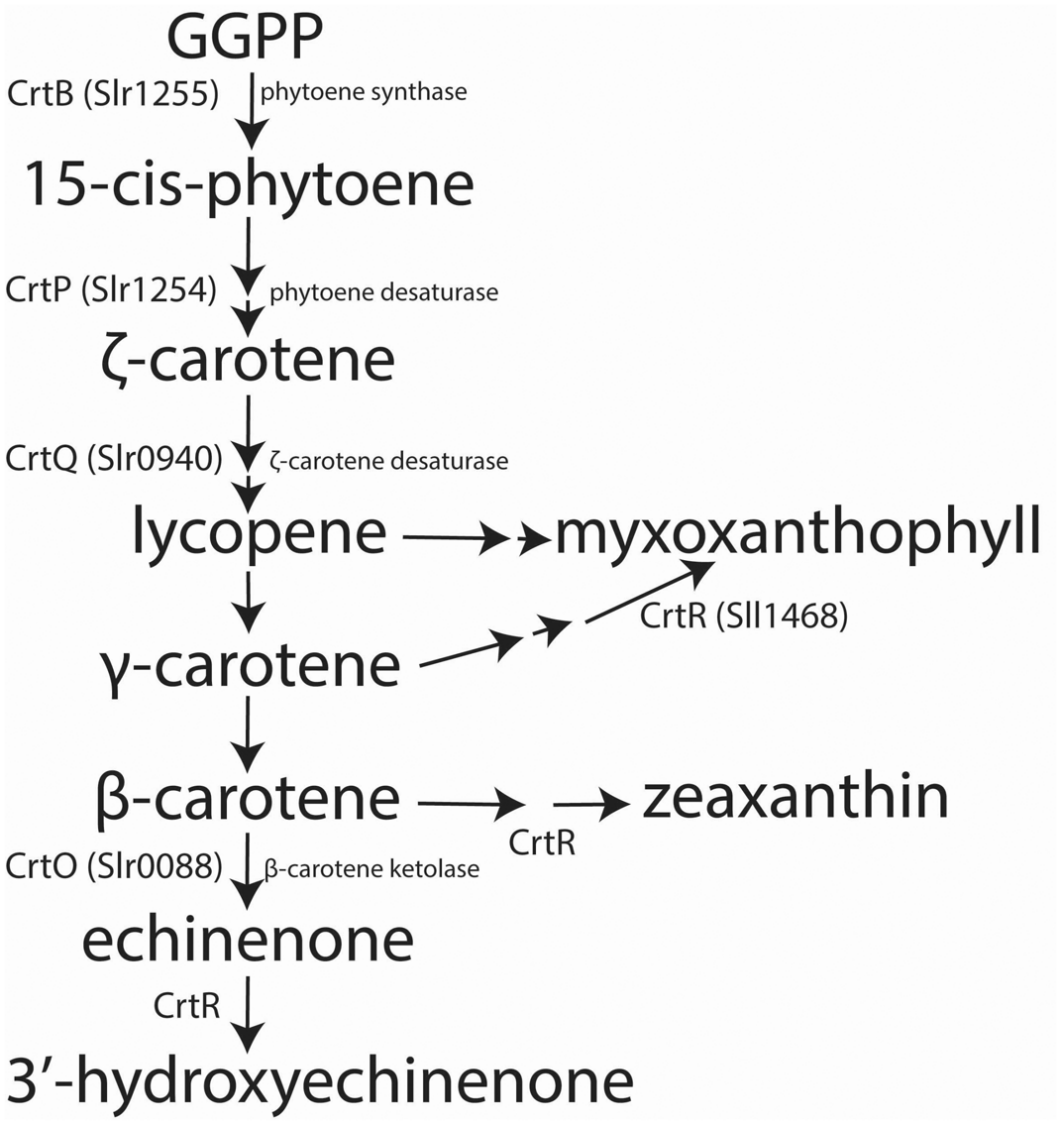
A simplified pathway for carotenoid biosynthesis in *Synehocystis*. Only enzymes with verified function are included.

The first committed step in carotenoid biosynthesis is the condensation of two molecules of geranylgeranyl pyrophosphate (GGPP) to form phytoene (see Fig 1) [13]. In *Synechocystis* this reaction is catalysed by phytoene synthase, encoded by the *crtB* (*slr1255*) gene [6]. Phytoene is then converted to various carotenes that are substrates for the synthesis of a variety of xanthophylls. Several enzymes have been identified and shown to be involved in the conversion of phytoene to β-carotene via lycopene. CrtP (Slr1254) is a phytoene desaturase synthesizing ζ-carotene, the deletion of which resulted in absence of β-carotene and its derivatives in *Synechocystis* [14]. ζ-carotene is further desaturated by CrtQ (Slr0940), a ζ-carotene desaturase that synthesizes lycopene [14–16]. Lycopene is the starting compound of various group modifications that produce a large variety of carotenoids with different physical properties. So far, four types of lycopene cyclase have been identified in different groups of bacteria, CrtL [17], CrtY [18,19], heterodimeric CrtY cyclases [20], and CruA/CruP [21]. Though cyclic carotenoids have been detected in *Synechocystis*, the enzyme responsible for cyclization of lycopene to β–carotene has not yet been positively identified. Sll0254, for instance, was previously reported to function as a lycopene cyclase/oxygenase but no evidence was found for its catalysing β–carotene formation directly [22,23]. β-carotene is further modified by enzymes such as CrtO [24] and CrtR [25], to produce echinenone and zeaxanthin respectively. The *crtO* (*slr0088*) gene, encoding the β-carotene ketolase, was previously inactivated in *Synechocystis*, with the resulting Δ*crtO* mutant being unable to synthesize echinenone [24].

β-carotene was found in the existing three-dimensional structures of photosynthetic complexes, with 12 molecules present in photosystem (PS)II [26], 22 in PSI [27] and one in the cytochrome (Cyt) b_6_f complex [28]. It is the major carotenoid species in *Synechocystis*, with an important role in photoprotection and shown to be required for assembly of PSII but not for that of PSI [29]. Other photoprotective mechanisms exist in cyanobacteria, one involving the down-regulation of photosynthesis by increasing thermal dissipation of the energy absorbed by the PSII antenna. The orange carotenoid protein (OCP), binding 3’-hydroxyechinenone as well as echinenone, is the key player in this photoprotective reaction in cyanobacteria [30,31].

There are two distinct inner membrane systems in *Synechocystis*, the plasma membrane (PM) and the thylakoid membrane (TM). PM can be further separated into lipid-rich “light” PM (PM1) and the major PM (PM2) fraction, which has the same density as the thylakoid membrane. The light PM fraction can be obtained by sucrose density gradient, while the major PM fraction can only be derived from TM by several steps of aqueous 2-phase polymer separations [32]. Even though the proteome of the different membrane fractions has been studied intensively, the study of carotenoid distribution is rare, especially at the level of subcellular membrane fractions. In this study, we focused on characterizing the carotenoid composition of different membranes in *Synechocystis* and locating the two important pathway steps catalysed by CrtQ and CrtO.

## Materials and Methods

### Strains and growth conditions

*Synechocystis* sp. PCC6803 wild type (WT) and mutants were grown in BG11 medium, in 1L glass bottles at 30°C, with sterile air bubbling and at a light intensity of 50 μE m^-2^ sec^-1^. Media were supplemented with 30 μg chloramphenicol ml^-1^ as required. Cell cultures with an OD_730_ between 1 and 1.5 were harvested by centrifugation at 8000 g and 4°C for 15 minutes, washed once in cold Lysis Buffer (20 mM potassium phosphate buffer, pH 7.8) and cell pellets were stored at -80°C until further use. For growth curve measurement, three independent WT and mutant cultures were grown to OD_730_ of 1.0, centrifuged at 3000 g for 15 min at room temperature and resuspended in fresh BG11 medium, without antibiotics, to a starting OD_730_ of 0.1. Cultures were incubated in a shaking incubator at 30°C, with constant overhead illumination at a light intensity of 50 μE m^-2^ sec^-1^ and shaking at 200 RPM.

### Construction of CrtQ-FLAG and CrtO-FLAG *Synechocystis* strains

Genomic DNA from *Synechocystis* was isolated [33] and the *crtQ* (*slr0940*) gene, together with up- and down-stream sequences of roughly 1 kbp was amplified from it by PCR (Phusion DNA polymerase, New England Biolabs; see S1 Table for primer sequences). This sequence was cloned into the pCR-Blunt II vector (Invitrogen) following manufacturers’ instructions, resulting in plasmid pCR-CrtQ. The 3xFLAG (hereafter, FLAG) epitope sequence was introduced in-frame at the 3’ terminal of *crtQ* gene, before its stop codon, by restriction-free cloning [34,35] using a synthetic dsDNA megaprimer (see S1 Table), resulting in plasmid pCR-CrtQ-FLAG. A chloramphenicol (Cm) resistance cassette, derived from plasmid pSK9 (a kind gift from Annegret Wilde), was amplified with hybrid primers (sequences in S1 Table) and likewise inserted to the vector pCR-CrtQ-FLAG by RF cloning. This constructed plasmid pCR-CrtQ-FLAG-Cm^R^ was transformed into the wild type *Synechocystis* cells as described [36]. The *crtO* gene (*slr0088*) was cloned and epitope-tagged using a similar strategy, using the primers described in S1 Table. Positive clones were confirmed by PCR using total genomic DNA as template (S1 Fig).

### Membrane fractionation

*Synechocystis* cell pellets from three independent 1 L cultures were washed once with chilled Lysis Buffer and resuspended in 1 mL of Lysis Buffer supplemented with protease inhibitors (Complete, EDTA-free, Roche). The cell suspension was lysed and membrane fraction preparation by sucrose density gradients/aqueous two-phase partitioning was performed according to previously established protocols [32,37]. Protein content was quantified by the Peterson method [38].

### Protein electrophoresis and Western blotting

5 μg membrane proteins from each strain were separated on 12% TGX precast SDS-PAGE gels (BioRad) or homemade 12% SDS-PAGE gels supplemented with 7M urea (for photosystem components). Western blots were performed as previously described [32], with antibodies for typical thylakoid membrane (PsaA and CP43, Agrisera) and plasma membrane proteins (KtrE, obtained from Nobuyuki Uozumi). Anti-FLAG M2 antibody was purchased from Sigma for detecting tagged proteins. To characterize the membrane association of CrtO and CrtQ, cells from three independent cultures were lysed as described above and total membranes and soluble fraction separated by ultracentrifugation at 200000 g, for 1 hr, at 4°C, as previously described [32]. 200 μg of total membranes were centrifuged at 200000 g, for 20 min at 4°C and resuspended in 50 μL of Extraction Buffer 8 (EB8; 20 mM Tricine, pH 8.0), Extraction buffer 12 (EB12; 20 mM CAPS, pH 12.0) or EB8 supplemented with 6M urea (EB8+U). Samples were incubated as previously described [39]. Briefly, samples were subjected to two freeze/thaw cycles (30 min at -80°C and 20 min at 20°C) after which they were centrifuged at 200000 g, for 20 min at 4°C. Pellets were resuspended in 50 μL of EB8 buffer and 5 μL of each sample (pellet or supernatant) were analysed by SDS-PAGE and western blotting (polyclonal PsbO antibodies were a kind gift from Peter Nixon). Each blot was repeated at least twice. To investigate whether CrtO and CrtQ were part of stable membrane complexes, pull-down experiments with anti-FLAG M2 resin (Sigma-Aldrich) and Clear Native (CN) gels were performed using total membrane samples, followed by western blots, according to previously established protocols [40].

### Carotenoid analysis by HPLC

Membrane fractions of *Synechocystis* cells were subjected to pigment extraction with two different methods, acetone:methanol (7/2, v/v) [41] and chloroform:methanol:water (1/1/0.8, v/v) [42]. In both cases the pigment extracts were dried under a stream of nitrogen and re-dissolved in ethanol. Both carotenoid preparations were separated by reversed-phase high-performance liquid chromatography (HPLC) at 15°C, with a flow rate of 1 mLmin^-1^ on a Purospher STAR RP-18 endcapped column, 150 mm x4.6 mm (Merck).

The pigment separation started with a 4 min isocratic elution with solvent A system of acetonitrile:methanol:Tris (0.1M, pH 8.0) (89/9.5/1.5, v/v), followed by a 2.5 min linear gradient of solvent B, methanol:hexane (4/1, v/v). Solvent B continued isocratically for 11.5 min before a linear gradient of solvent A for 1 min, and a final isocratic elution with solvent A for 9 min. The total run time was 28 min. Carotenoids were detected at 440 nm. Pigments were identified by comparison of the known carotenoid composition of *Synechocystis* [43], the HPLC retention times, absorption spectra and commercially available zeaxanthin, β-carotene (Extrasynthèse, www.extrasynthese.com), myxoxanthophyll and echinenone (DHI www.labproducts.dhigroup.com) standards. The concentration of zeaxanthin, β-carotene, myxoxanthphyll and echinenone was quantified based on the peak area of known standards (Extrasynthèse and DHI). The minor component γ-carotene was quantified as β-carotene and hydroxyechinenone and cryptoxanthin were quantitated as zeaxanthin. Three independent membrane preparations were used for the carotenoid analysis, and each sample was analyzed typically 3 times. The handling of pigment samples was performed in dim light.

### Pigment composition analysis by absorption scanning

Pigment composition was compared between WT and FLAG-tagged strains by performing absorption scans on exponentially grown cells in BG11 medium as previously described [44]. Cells were diluted with BG11 to an OD_730_ of ≈0.22 and absorption was measured from 380 to 730 nm using a Cary 300 Bio UV-Visible Spectrophotometer (Varian). The spectra obtained (S3 Fig) were normalized to the absorption at 730 nm.

## Results

### Light plasma membrane has the highest carotenoid/protein ratio, while the heavy plasma membrane has the lowest

**Fig 2:**
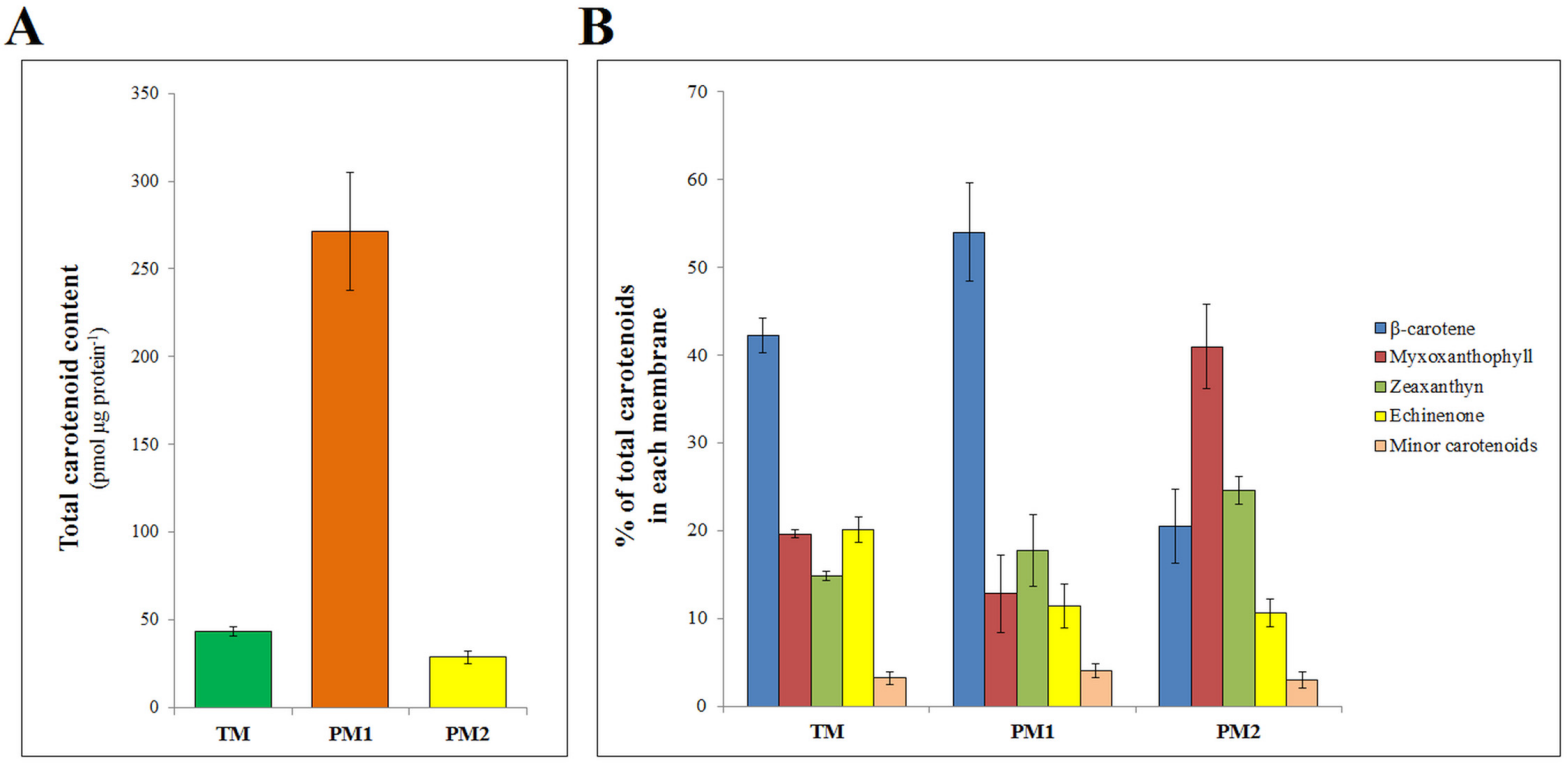
Carotenoid distribution in *Synechocystis* membranes. Total carotenoid amount in purified PM1, PM2 and TM (A). Relative amount of different carotenoids in each membrane (B). Data represent means ± standard deviations for carotenoids in membranes from three independent cultures. For results of statistical analysis, please refer to S2 Table.

Carotenoid extraction from PM1, PM2 and TM of wild type cells was carried out with two different extraction methods. Similar results were obtained with both methods and included in the mean of each independent sample. Substantial differences were observed between different membranes, with the total content of carotenoids on a protein basis being highest in PM1, while in TM and PM2 there were only 16% and 11% respectively of the PM1 carotenoid content (Fig 2A). The carotenoid composition analyses revealed that each membrane has the same kind of carotenoids though in different proportions (Fig 2B). For instance, in PM1, 54% of the total carotenoid content is comprised of β-carotene, with myxoxanthophyll, zeaxanthin, echinenone and other minor carotenoids. This character is similar to that of TM (the most abundant membrane in *Synechocystis*), which also contains mostly β-carotene, albeit in a lower concentration (42%). PM2, on the other hand, has a different composition in comparison to other membranes, with 41% of myxoxanthophyll, 25% of zeaxanthin and 20% of β-carotene.

### CrtQ and CrtO are more concentrated in plasma membrane than thylakoid membrane

**Fig 3:**
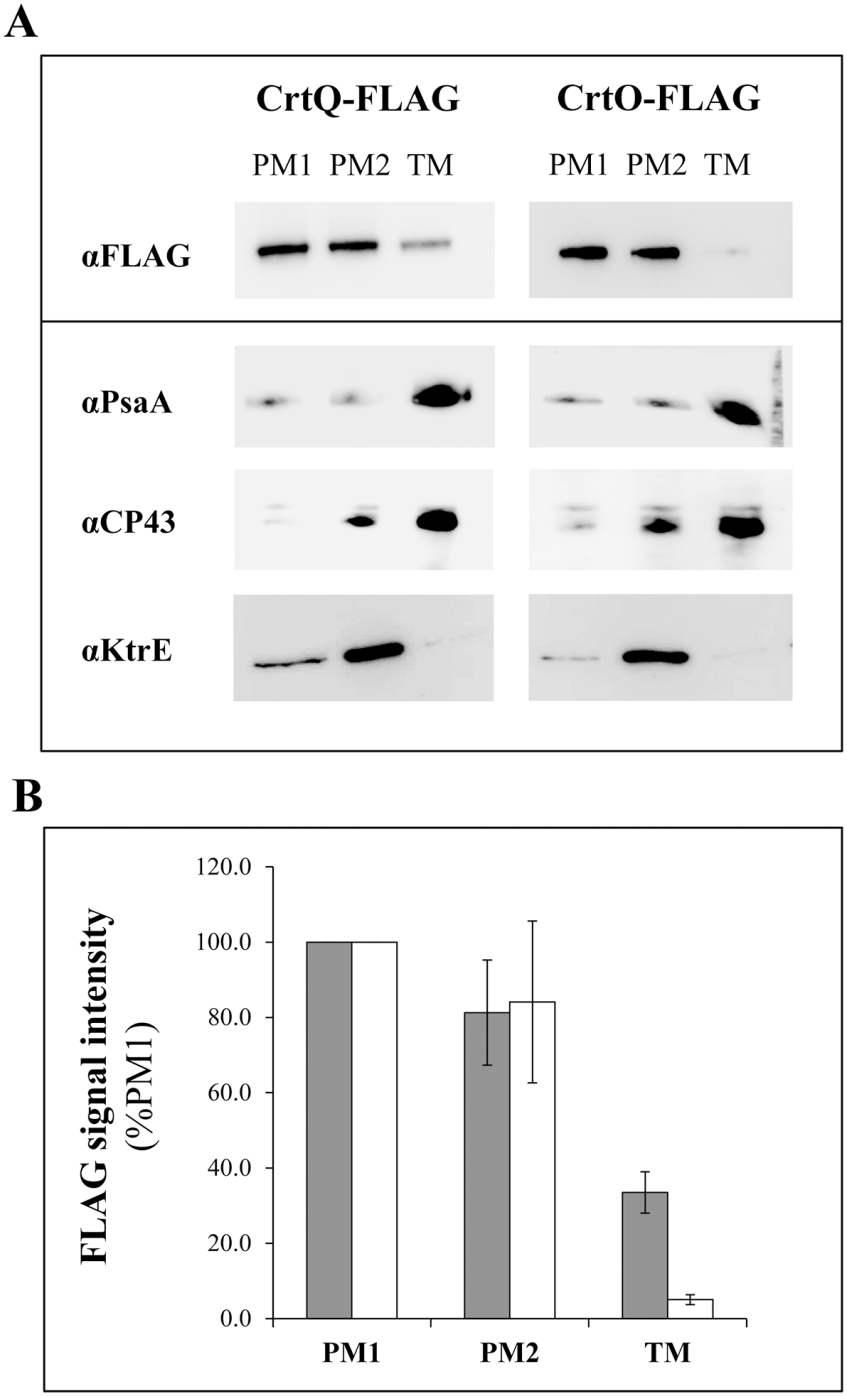
CrtQ and CrtO localization. Western blots analyses of purified PM1, PM2 and TM from CrtQ-FLAG and CrtO-FLAG strains. Exposure times were 4 and 180 seconds respectively (A). Quantifications of CrtQ (grey) and CrtO (white) in each membrane, based on four independent western blots (B). Data represent means ± standard deviations for four independent quantifications. For results of statistical analysis, please refer to S2 Table.

To understand the cause for this unequal carotenoid distribution in *Synechocystis* membranes, the distribution of enzymes involved in carotenoid biosynthesis was further investigated. Two of these enzymes, CrtQ and CrtO, were chosen due to their well proven functions. As suitable antibodies are unavailable, 3xFLAG epitopes were added to the C-terminal end of each protein, which were under the control of their original promoters and in the original loci. The strains thus obtained did not show measurable pigment and growth differences (see S2 and S3 Figs). PM1, PM2 and TM were isolated from CrtQ-FLAG and CrtO-FLAG cells and the purity of the membrane fractions were confirmed with antibodies against PsaA, CP43 and KtrE (Fig 3A). Anti-FLAG blots showed single specific band in both strains, yet with slight differences regarding their distribution. In the case of CrtQ, the highest concentration of the FLAG signal could be detected in PM1 and PM2, with similar amounts in these two membranes (Fig 3). At the same time, only about 30% of the amount in PM1 could also be found in TM. On the other hand, our data showed that CrtO is strongly concentrated in PM, with similar distribution between PM1 and PM2. To get a similar intensity for both proteins, roughly 50 times longer exposure was needed for CrtO (3 min vs 4 sec), indicating that CrtO was less abundant than CrtQ.

### No large complexes containing CrtQ or CrtO proteins observed in *Synechocystis* membranes

**Fig 4:**
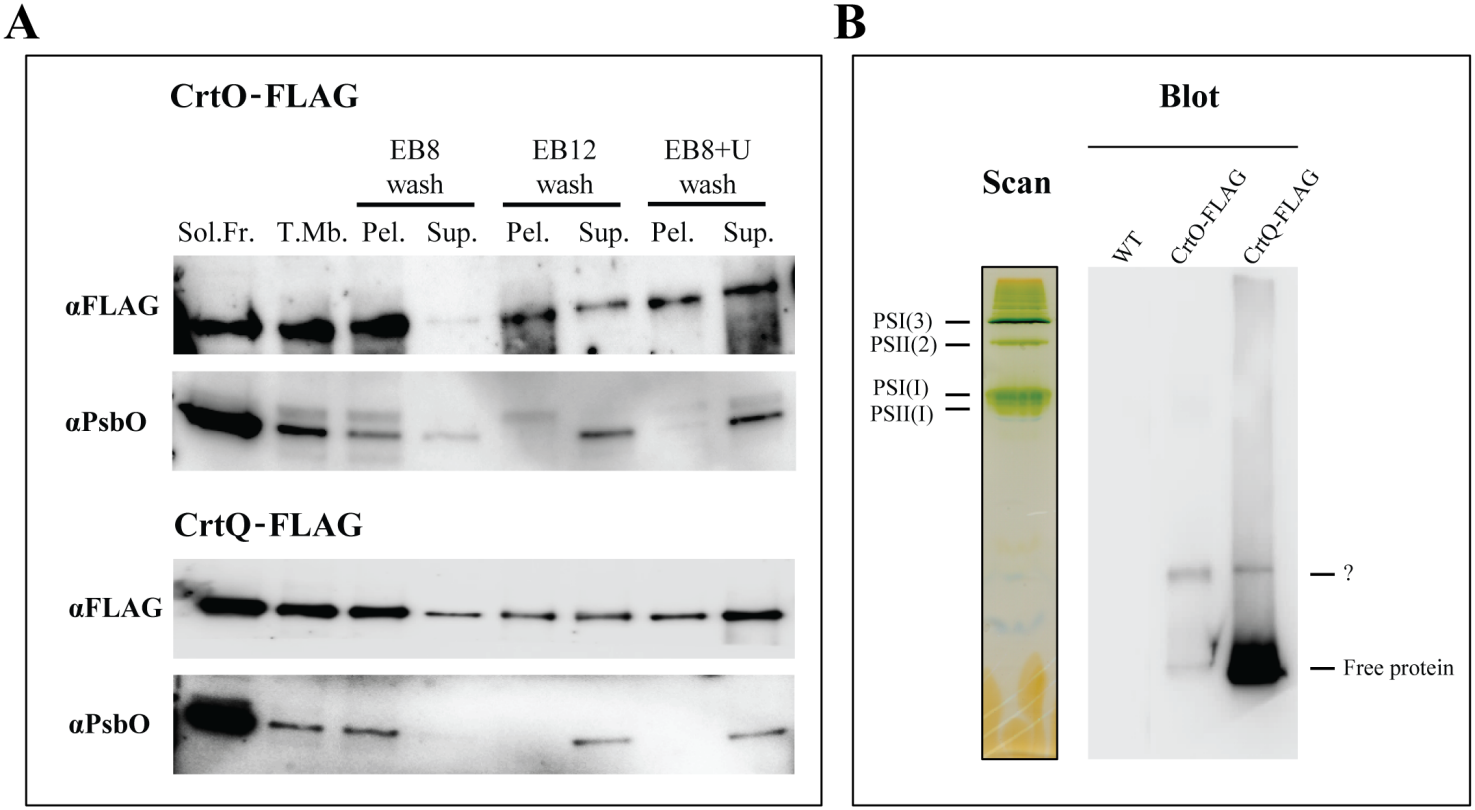
Membrane binding analyses of CrtQ-FLAG and CrtO-FLAG. Western blots of soluble fractions vs. total membranes (5 μg each) isolated from both strains and the effect of different washes on CrtO and CrtQ membrane association. Total membranes were washed with EB8, EB12 or EB8+U and soluble (“Sol.”) and pellet (“Pel.”) fractions were generated by ultracentrifugation. Each lane was loaded with equal volumes of (resuspended) pellet or supernatant fractions. Three independent cultures were tested in this manner. (A). Western blots analysis of anti-FLAG resin eluates from 2% DDM-solubilized total membranes of WT, CrtO-FLAG and CrtQ-FLAG cells, separated on 4–18% Clear Native–PAGE (B). Colour scan of CN-PAGE gel for WT total membrane showing the relative migration of several known complexes.

Both CrtQ and CrtO are predicted to be soluble proteins, with some hydrophobic regions in the N-terminal, by online softwares TOPCONS (www.topcons.net) and TMHMM (http://www.cbs.dtu.dk/services/TMHMM/). These proteins are involved in the synthesis of carotenoid molecules and it is expected, from the hydrophobic nature of their products, that they should strongly associate to the membrane. Total membranes were washed with both high pH and high concentrations of urea, so as to remove membrane associated proteins, and anti-FLAG western blots of the resulting fractions showed that, though also present in the soluble fraction, neither membrane-associated CrtO nor CrtQ could be completely removed by either high pH or urea treatment, in opposition to the luminal PSII subunit PsbO (Fig 4A).

To clarify whether these proteins form part of large protein complexes, associate to the photosystems or are involved in other integral membrane complexes in *Synechocystis*, we attempted to isolate CrtO- and CrtQ-containing complexes by pull-down with anti-FLAG resin and analysis of the resulting eluates in CN-PAGE/western blots. In both cases we were unable to detect high molecular weight complexes containing either CrtO or CrtQ (Fig 4B). CrtQ could be seen mostly in the form of free protein (Fig 4B). CrtO, though the signal is much weaker, could also be seen to migrate in the low molecular range of the gel, while no signal was observed in the WT pulldown sample. A second slower migrating band could be observed in both cases, though its nature could not be determined by western blot at this moment.

## Discussions

### Where does carotenoid biosynthesis occur in Synechocystis?

Carotenoid biosynthesis in cyanobacteria is not as well characterized as in plants. In *Synechocystis*, for instance, only a handful of enzymes (CrtB, CrtP, CrtQ, CrtO and CrtR) have had their function experimentally proven (Fig 1) [6,14–16,24]. Furthermore, their respective intracellular distribution has not yet been established. Our results show that CrtQ, one of the enzymes involved in the early steps of β-carotene formation, has a higher local concentration in PM than in TM (Fig 3). The carotenoid quantification data showed highest pigment/protein ratios in PM1 (Fig 2A). Since TM is the most abundant membrane and PM1 only constitutes a very small part, the majority of carotenoids present in the cell exist in the TM. At the same time, over 40% of the total carotenoid accumulated in TM is β-carotene (Fig 2B). The large proportion of this pigment in the light plasma membrane fraction (PM1) and the high local concentration of the enzyme (CrtQ) responsible for synthesis of its precursor could reflect a local heterogeneity in the plasma membrane. Lycopene does not accumulate to any measurable extent in *Synechocystis*; therefore, the conversion between lycopene and β-carotene must be fast and complete. As such, though we are only able to locate the enzyme responsible for lycopene synthesis, it seems logical to consider that PM1 could be the major synthesis site of lycopene and, due to this fast conversion between the two, of β-carotene as well.

In addition, CrtO, which is the enzyme catalysing the formation of echinenone and 3-hydroxyechinenone in the late stage of carotenoid synthesis, is also strongly concentrated in PM but not in TM (Fig 3). Both echinenone and 3’-hydroxyechinenone loading are important for functional OCP [30,31]. It was previously shown that OCP strongly interacts with the thylakoids, acting as a quencher to dissipate the excess energy arriving at the reaction centres [45]. Our results indicate that a higher local synthesis of echinenone and 3’-hydroxyechinenone should take place in PM. Whether the pigment loading to OCP happens in PM or echinenone/3’-hydroxiechinenone diffuse to TM prior to OCP loading still remains to be ascertained.

Owing to the scarcity of data on the identity of carotenoid synthesis enzymes in *Synechocystis*, we chose to study the location of these two particular enzymes. CrtQ is responsible for the synthesis of lycopene, the step just prior to conversion to β-carotene, thus serving as a means to locate early carotenoid biosynthesis steps. CrtO, on the other hand, catalyses the synthesis of a pigment after the branching point of the carotenoid pathway (Fig 1), essential for non-photochemical quenching. However, both of these enzymes seem to be much more locally concentrated in the plasma membrane and, together with the strikingly high carotenoid/protein ratio in PM1, it is tempting to point to PM, and especially to PM1, as being the major site of carotenoid synthesis in *Synechocystis*. CrtO, in particular, is especially concentrated in the PM fractions (Fig 3). Further research will be required to unambiguously identify other enzymes within the pathway (for instance, the glycosyltransferase required for myxoxanthophyll synthesis), at which point we shall be able to confirm out hypothesis. However, an interesting parallel can be established here with another cyanobacterium, *Gloeobacter violaceus*. This unusual cyanobacterium does not have separate thylakoid membranes but, instead, discrete domains within its plasma membrane where photosynthetic complexes are located [46]. This “green fraction” can be isolated from the “orange fraction” by biochemical methods and the composition of both fractions was previously studied in detail. It was concluded that these different fractions or domains are formed by lateral heterogeneity and phase transitions induced by their very different protein and pigment composition, rather than differences in lipid species, as would be the case of lipid rafts. The “orange fraction” contains more carotenoids than the “green fraction”, which had 20 times more chlorophyll but only 4% of the carotenoid content [46]. Furthermore, an enzyme of the carotenoid biosynthesis pathway—phytoene desaturase or CrtI—was found exclusively in the “orange fraction” [46] and it was suggested that the carotenoid synthesis-related proteins should therefore accumulate in the “orange fraction”. It is tempting to see the PM1 fraction of *Synechocystis* as being very similar to this “orange fraction” of *G*. *violaceus*—laterally heterogeneous fractions of the membrane where enzymes responsible for carotenoid synthesis and their respective products accumulate and where, perhaps, the synthesis rate of these compounds is the highest.

### β-carotene biosynthesis does not directly interact with the photosynthetic complexes

Carotenoids are an intrinsic part of photosystem structures, as previously mentioned, and it was proposed that their synthesis regulates PSII assembly [6]. As such, it was important to understand whether CrtQ would form membrane complexes, either with PSII assembly precursors or with other (putative) proteins, acting as mediators in PSII β-carotene loading. Our results show that CrtQ exists in a considerable amount in TM (30% of the amount in PM, Fig 3), but it does not seem to form high molecular weight complexes with PSII or other assembly intermediates, being present mostly as a free protein (Fig 4B). A weak band could also be seen above the free CrtQ band, though its composition is unknown for the moment. This is in opposition to what is described in higher plants, where the carotenoid synthesis enzymes apparently form a large membrane-associated complex [47] Thus, it is likely that lycopene formed by CrtQ flows freely into the membrane, from where it is then inserted into the photosystem complexes when required. We were also unable to identify any large complexes containing CrtO. Though the association of these two proteins to the membrane is quite strong (see Fig 4A), so far we were unable to positively identify interacting membrane protein partners that would stabilize them in a high molecular weight complex. As such, it may be that their association to the membrane is mostly dependent on hydrophobic interactions, either with the lipid or pigment components.

## Supporting Information

S1 Fig 0.8% agarose gel of segregation screening PCR for FLAG-tagged CrtO and CrtQ *Synechocystis* strains. Segregation screening PCR was performed using primers indicated in S1 Table.
(TIF)

S2 Fig Growth curves for WT and FLAG-tagged CrtO and CrtQ *Synechocystis* strains. OD_730_ data points are the average of three independent cultures, with error bars representing the standard deviation.
(TIF)

S3 Fig Representative whole cell absorption scans of WT and FLAG-tagged CrtO and CrtQ *Synechocystis* strains. (TIF)

S4 Fig HPLC chromatogram of a typical PM1 pigment extract. Peaks were detected by absorbance at 478 nm. Labelled peaks correspond to myxoxantophyll (1), zeaxanthin (2), hydroxyechinenone (3), echinenone (4), cryptoxanthin (5), β-carotene (6) and γ-carotene (7). Chlorophyll retention time under these conditions was 11.2 minutes (chlorophyll peak is not visible at 478 nm).
(TIF)

S1 Table Primers used in construction of FLAG-tagged CrtO and CrtQ *Synechocystis* strains. (DOCX)

S2 Table P-values for Student’s t-test analysis of quantifications shown in Figs 2 and 3. P<0.05 was considered significant.
(XLSX)

## References

[1] Gruszecki WI, Strzalka K (2005) Carotenoids as modulators of lipid membrane physical properties. Biochim Biophys Acta 1740: 108–115. 15949676 10.1016/j.bbadis.2004.11.015

[2] Schafer L, Vioque A, Sandmann G (2005) Functional in situ evaluation of photosynthesis-protecting carotenoids in mutants of the cyanobacterium *Synechocystis* PCC6803. J Photochem Photobiol B 78: 195–201. 15708516 10.1016/j.jphotobiol.2004.11.007

[3] Zhu YH, Graham JE, Ludwig M, Xiong W, Alvey RM, Shen GZ, et al (2010) Roles of xanthophyll carotenoids in protection against photoinhibition and oxidative stress in the cyanobacterium *Synechococcus* sp strain PCC 7002. Arch Biochem Biophys 504: 86–99. 10.1016/j.abb.2010.07.007 20638360

[4] Szabo I, Bergantino E, Giacometti GM (2005) Light and oxygenic photosynthesis: energy dissipation as a protection mechanism against photo-oxidation. EMBO Rep 6: 629–634. 15995679 10.1038/sj.embor.7400460 PMC1369118

[5] Cogdell RJ, Howard TD, Bittl R, Schlodder E, Geisenheimer I, Lubitz W (2000) How carotenoids protect bacterial photosynthesis. Philos Trans R Soc Lond B Biol Sci 355: 1345–1349. 11127989 10.1098/rstb.2000.0696 PMC1692869

[6] Sozer O, Komenda J, Ughy B, Domonkos I, Laczko-Dobos H, Malec P, et al (2010) Involvement of carotenoids in the synthesis and assembly of protein subunits of photosynthetic reaction centers of *Synechocystis* sp PCC 6803. Plant Cell Physiol 51: 823–835. 10.1093/pcp/pcq031 20231245

[7] Takaichi S, Maoka T, Masamoto K (2001) Myxoxanthophyll in *Synechocystis* sp PCC 6803 is myxol 2 '-dimethyl-fucoside, (3R,2 ' S)-myxol 2 '-(2,4-di-O-methyl-alpha-L-fucoside), not rhamnoside. Plant Cell Physiol 42: 756–762. 11479383 10.1093/pcp/pce098

[8] Takaichi S, Mochimaru M, Maoka T (2006) Presence of free myxol and 4-hydroxymyxol and absence of myxol glycosides in *Anabaena variabilis* ATCC 29413, and proposal of a biosynthetic pathway of carotenoids. Plant Cell Physiol 47: 211–216. 16338959 10.1093/pcp/pci236

[9] Takaichi S, Mochimaru M, Maoka T, Katoh H (2005) Myxol and 4-ketomyxol 2 '-fucosides, not rhamnosides, from *Anabaena* sp PCC 7120 and *Nostoc punctiforme* PCC 73102, and proposal for the biosynthetic pathway of carotenoids. Plant Cell Physiol 46: 497–504. 15695449 10.1093/pcp/pci049

[10] Masamoto K, Furukawa K (1997) Accumulation of zeaxanthin in cells of the cyanobacterium, *Synechococcus* sp. strain PCC 7942 grown under high irradiance. J Plant Physiol 151: 257–261.

[11] Steiger S, Schafer L, Sandmann G (1999) High-light-dependent upregulation of carotenoids and their antioxidative properties in the cyanobacterium *Synechocystis* PCC 6803. J Photochem Photobiol B 52: 14–18.

[12] Miller SR, Martin M, Touchton J, Castenholz RW (2002) Effects of nitrogen availability on pigmentation and carbon assimilation in the cyanobacterium *Synechococcus* sp strain SH-94-5. Arch Microbiol 177: 392–400. 11976748 10.1007/s00203-002-0404-8

[13] Martinezferez I, Fernandezgonzalez B, Sandmann G, Vioque A (1994) Cloning and expression in *Escherichia coli* of the gene coding for phytoene synthase from the cyanobacterium *Synechocystis* sp. PCC6803. Biochim Biophys Acta 1218: 145–152. 8018713 10.1016/0167-4781(94)90003-5

[14] Bautista JA, Tracewell CA, Schlodder E, Cunningham FX, Brudvig GW, Diner BA (2005) Construction and characterization of genetically modified *Synechocystis* sp. PCC 6803 Photosystem II core complexes containing carotenoids with shorter pi-conjugation than beta-carotene. J Biol Chem 280: 38839–38850. 16159754 10.1074/jbc.M504953200

[15] Mohamed HE, van de Meene AML, Roberson RW, Vermaas WFJ (2005) Myxoxanthophyll is required for normal cell wall structure and thylakoid organization in the cyanobacterium *Synechocystis* sp strain PCC 6803. J Bacteriol 187: 6883–6892. 16199557 10.1128/JB.187.20.6883-6892.2005 PMC1251633

[16] Breitenbach J, Fernandez-Gonzalez B, Vioque A, Sandmann G (1998) A higher-plant type zeta-carotene desaturase in the cyanobacterium *Synechocystis sp*. PCC6803. Plant Mol Biol 36: 725–732. 9526505 10.1023/a:1005997405431

[17] Misawa N, Nakagawa M, Kobayashi K, Yamano S, Izawa Y, Nakamura K, et al (1990) Elucidation of the *Erwinia uredovora c*arotenoid biosynthetic pathway by functional analysis of gene products expressed in *Escherichia coli* . J Bacteriol 172: 6704–6712. 2254247 10.1128/jb.172.12.6704-6712.1990 PMC210783

[18] Cunningham FX, Sun ZR, Chamovitz D, Hirschberg J, Gantt E (1994) Molecular structure and enzymatic function of lycopene cyclase from the cyanobacterium *Synechococcus* sp. strain PCC7942. Plant Cell 6: 1107–1121. 7919981 10.1105/tpc.6.8.1107 PMC160505

[19] Stickforth P, Steiger S, Hess WR, Sandmann G (2003) A novel type of lycopene epsilon-cyclase in the marine cyanobacterium *Prochlorococcus marinus* MED4. Arch Microbiol 179: 409–415. 12712234 10.1007/s00203-003-0545-4

[20] Krubasik P, Sandmann G (2000) A carotenogenic gene cluster from *Brevibacterium linens* with novel lycopene cyclase genes involved in the synthesis of aromatic carotenoids. Mol Gen Genet 263: 423–432. 10821176 10.1007/s004380051186

[21] Maresca JA, Graham JE, Wu M, Eisen JA, Bryant DA (2007) Identification of a fourth family of lycopene cyclases in photosynthetic bacteria. Proceedings of the National Academy of Sciences of the United States of America 104: 11784–11789. 17606904 10.1073/pnas.0702984104 PMC1905924

[22] Mohamed HE, Vermaas WFJ (2006) S110254 (CrtL(diox)) is a bifunctional lycopene cyclase/dioxygenase in cyanobacteria producing myxoxanthophyll. J Bacteriol 188: 3337–3344. 16621828 10.1128/JB.188.9.3337-3344.2006 PMC1447463

[23] Maresca JA, Graham JE, Wu M, Eisen JA, Bryant DA (2007) Identification of a fourth family of lycopene cyclases in photosynthetic bacteria. Proc Natl Acad Sci U S A 104: 11784–11789. 17606904 10.1073/pnas.0702984104 PMC1905924

[24] Fernandez-Gonzalez B, Sandmann G, Vioque A (1997) A new type of asymmetrically acting beta-carotene ketolase is required for the synthesis of echinenone in the cyanobacterium *Synechocystis* sp. PCC 6803. J Biol Chem 272: 9728–9733. 9092504 10.1074/jbc.272.15.9728

[25] Lagarde D, Vermaas W (1999) The zeaxanthin biosynthesis enzyme beta-carotene hydroxylase is involved in myxoxanthophyll synthesis in *Synechocystis* sp. PCC6803. FEBS Lett 454: 247–251. 10431816 10.1016/s0014-5793(99)00817-0

[26] Guskov A, Gabdulkhakov A, Broser M, Glockner C, Hellmich J, Kern J, et al (2010) Recent progress in the crystallographic studies of Photosystem II. Chemphyschem 11: 1160–1171. 10.1002/cphc.200900901 20352642

[27] Grotjohann I, Fromme P (2005) Structure of cyanobacterial Photosystem I. Photosynth Res 85: 51–72. 15977059 10.1007/s11120-005-1440-4

[28] Baniulis D, Yamashita E, Whitelegge JP, Zatsman AI, Hendrich MP, Hasan SS, et al (2009) Structure-function, stability, and chemical modification of the cyanobacterial cytochrome b(6)f complex from *Nostoc* sp PCC 7120. J Biol Chem 284: 9861–9869. 10.1074/jbc.M809196200 19189962 PMC2665108

[29] Masamoto K, Hisatomi S, Sakurai I, Gombos Z, Wada H (2004) Requirement of carotene isomerization for the assembly of photosystem II in *Synechocystis* sp PCC 6803. Plant Cell Physiol 45: 1325–1329. 15509857 10.1093/pcp/pch144

[30] Punginelli C, Wilson A, Routaboul JM, Kirilovsky D (2009) Influence of zeaxanthin and echinenone binding on the activity of the Orange Carotenoid Protein. Biochim Biophys Acta 1787: 280–288. 10.1016/j.bbabio.2009.01.011 19366615

[31] Wilson A, Kinney JN, Zwart PH, Punginelli C, D'Haene S, Perreau F, et al (2010) Structural determinants underlying photoprotection in the photoactive Orange Carotenoid Protein of cyanobacteria. J Biol Chem 285: 18364–18375. 10.1074/jbc.M110.115709 20368334 PMC2881762

[32] Selao TT, Zhang LF, Arioz C, Wieslander A, Norling B (2014) Subcellular localization of monoglucosyldiacylglycerol synthase in *Synechocystis* sp. PCC6803 and its unique regulation by lipid environment. PLoS One 9. 10.1371/journal.pone.0088153 PMC3916417 24516600

[33] Volkmer T, Schneider D, Bernat G, Kirchhoff H, Wenk SO, Rogner M (2007) Ssr2998 of *Synechocystis* sp PCC 6803 is involved in regulation of cyanobacterial electron transport and associated with the cytochrome b(6)f complex. J Biol Chem 282: 3730–3737. 17166849 10.1074/jbc.M604948200

[34] Bond SR, Naus CC (2012) RF-Cloning.org: an online tool for the design of restriction-free cloning projects. Nucleic Acids Res 40: W209–W213. 10.1093/nar/gks396 22570410 PMC3394257

[35] van den Ent F, Lowe J (2006) RF-cloning: A restriction-free method for inserting target genes into plasmids. J Biochem Biophys Methods 67: 67–74. 16480772 10.1016/j.jbbm.2005.12.008

[36] Eaton-Rye JJ (2011) Construction of gene interruptions and gene deletions in the cyanobacterium *Synechocystis* sp. strain PCC 6803. Methods Mol Biol 684: 295–312. 10.1007/978-1-60761-925-3_22 20960137

[37] Huang F, Parmryd I, Nilsson F, Persson AL, Pakrasi HB, Andersson B, et al (2002) Proteomics of *Synechocystis* sp strain PCC 6803—identification of plasma membrane proteins. Mol Cell Proteomics 1: 956–966. 12543932 10.1074/mcp.m200043-mcp200

[38] Peterson GL (1977) Simplification of protein assay method of Lowry et al. which is more generally applicable. Anal Biochem 83: 346–356. 603028 10.1016/0003-2697(77)90043-4

[39] Boehm M, Nield J, Zhang P, Aro EM, Komenda J, Nixon PJ (2009) Structural and mutational analysis of band 7 proteins in the cyanobacterium *Synechocystis* sp. strain PCC 6803. J Bacteriol 191: 6425–6435. 10.1128/JB.00644-09 19684140 PMC2753028

[40] Chidgey JW, Linhartova M, Komenda J, Jackson PJ, Dickman MJ, Canniffe DP, et al (2014) A cyanobacterial chlorophyll synthase-HliD complex associates with the Ycf39 protein and the YidC/Alb3 insertase. Plant Cell 26: 1267–1279. 10.1105/tpc.114.124495 24681617 PMC4001383

[41] Domonkos I, Malec P, Laczko-Dobos H, Sozer O, Klodawska K, Wada H, et al (2009) Phosphatidylglycerol depletion induces an increase in myxoxanthophyll biosynthetic activity in *Synechocystis sp*. PCC 6803 cells. Plant Cell Physiol 50: 374–382. 10.1093/pcp/pcn204 19131356

[42] Bligh EG, Dyer WJ (1959) A rapid method of total lipid extraction and purification. Can J Biochem Physiol 37: 911–917. 13671378 10.1139/o59-099

[43] Maresca JA, Graham JE, Bryant DA (2008) The biochemical basis for structural diversity in the carotenoids of chlorophototrophic bacteria. Photosynth Res 97: 121–140. 10.1007/s11120-008-9312-3 18535920

[44] Peramuna A, Morton R, Summers ML (2015) Enhancing alkane production in cyanobacterial lipid droplets: a model platform for industrially relevant compound production. Life (Basel) 5: 1111–1126. 10.3390/life5021111 25821934 PMC4500132

[45] Wilson A, Ajlani G, Verbavatz JM, Vass I, Kerfeld CA, Kirilovsky D (2006) A soluble carotenoid protein involved in phycobilisome-related energy dissipation in cyanobacteria. Plant Cell 18: 992–1007. 16531492 10.1105/tpc.105.040121 PMC1425857

[46] Rexroth S, Mullineaux CW, Ellinger D, Sendtko E, Rogner M, Koenig F (2011) The plasma membrane of the cyanobacterium *Gloeobacter violaceus* contains segregated bioenergetic domains. Plant Cell 23: 2379–2390. 10.1105/tpc.111.085779 21642550 PMC3160022

[47] Lopez AB, Yang Y, Thannhauser TW, Li L (2008) Phytoene desaturase is present in a large protein complex in the plastid membrane. Physiol Plant 133: 190–198. 10.1111/j.1399-3054.2008.01058.x 18298413

