# Supplementary figures and images for: Subcellular Localization of Carotenoid Biosynthesis in Synechocystis sp. PCC 6803
Source: PLoS One. 2015 Jun 17;10(6):e0130904. doi: 10.1371/journal.pone.0130904 (PMC4470828; doi:10.1371/journal.pone.0130904)

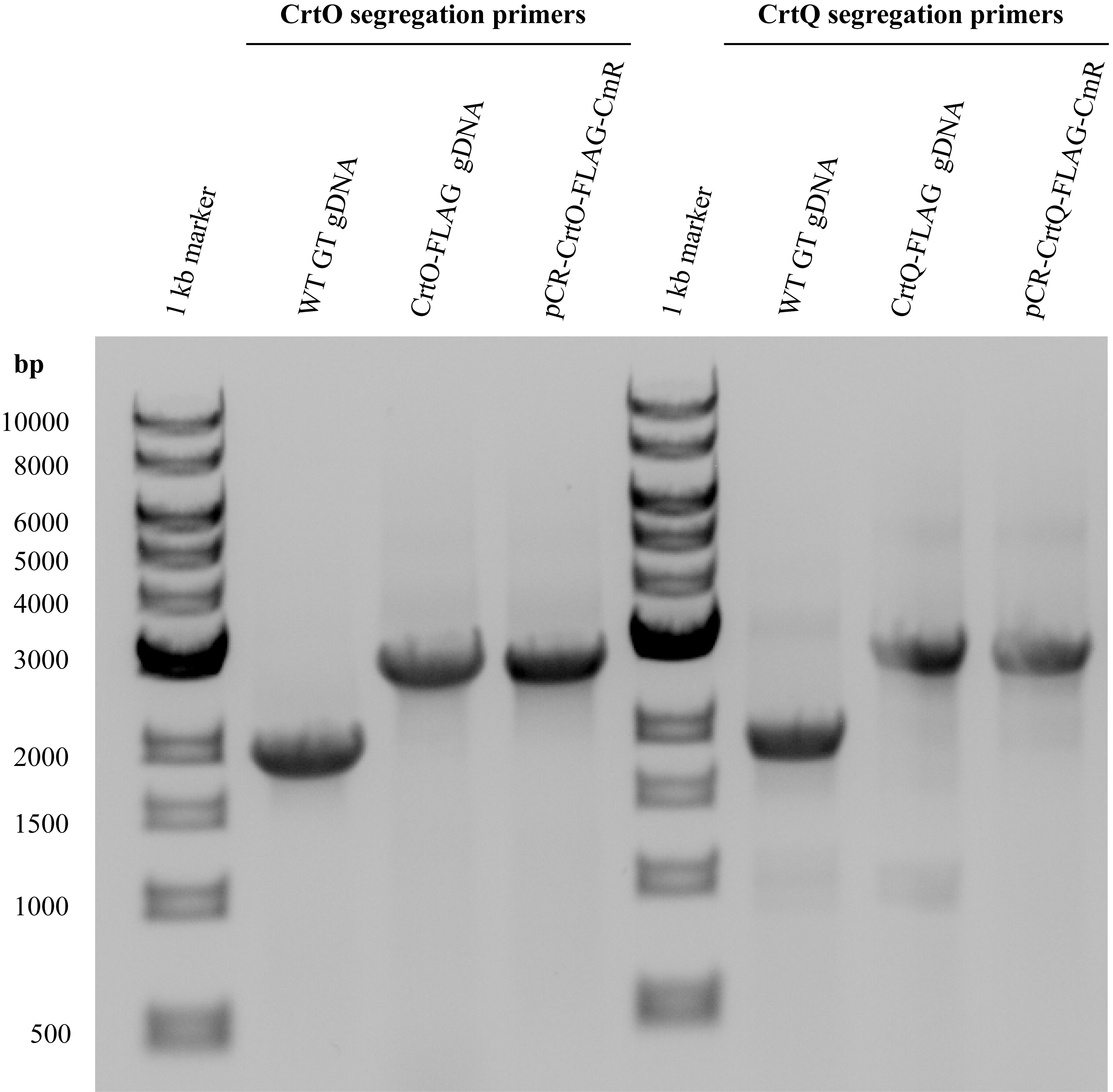

Supplement: S1 Fig — Segregation screening PCR was performed using primers indicated in S1 Table. (TIF) [file pone.0130904.s001.tif]

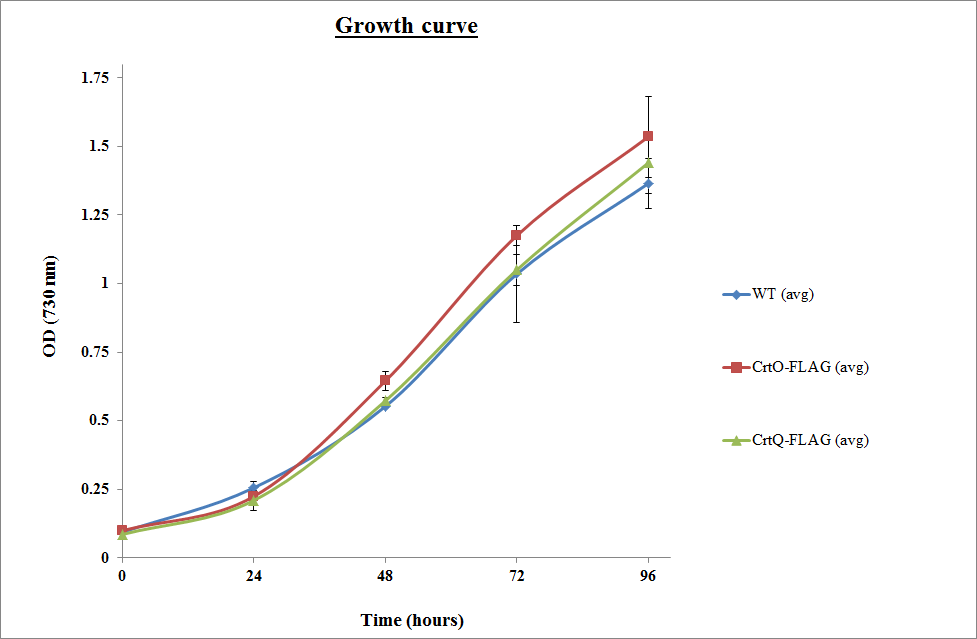

Supplement: S2 Fig — OD730 data points are the average of three independent cultures, with error bars representing the standard deviation. (TIF) [file pone.0130904.s002.tif]

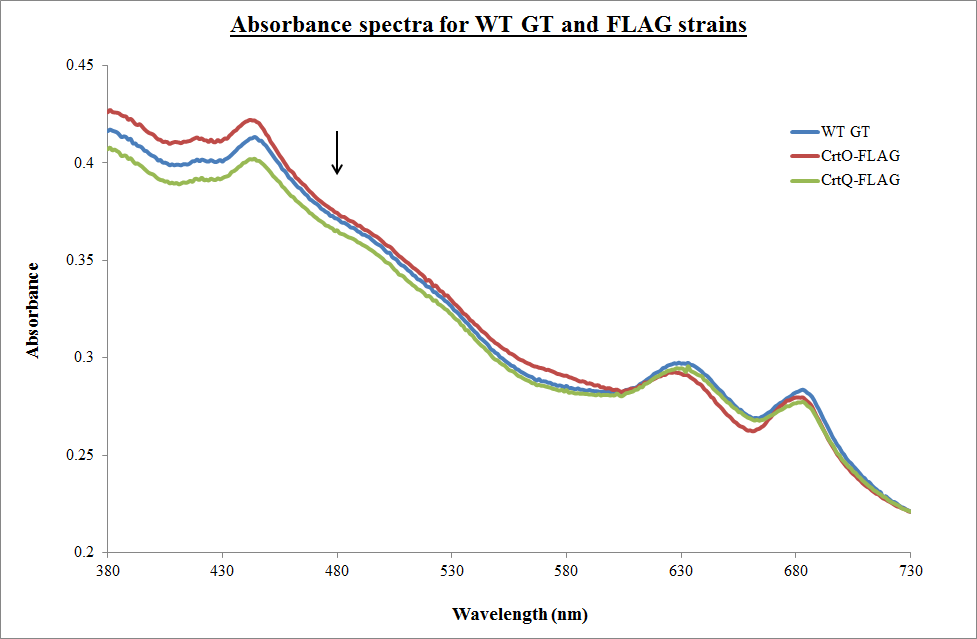

Supplement: S3 Fig — (TIF) [file pone.0130904.s003.tif]
