# Supplementary material for: Subcellular Localization of Carotenoid Biosynthesis in Synechocystis sp. PCC 6803
Source: PLoS One. 2015 Jun 17;10(6):e0130904. doi: 10.1371/journal.pone.0130904 (PMC4470828; doi:10.1371/journal.pone.0130904)
Supplement: S1 Table — (DOCX) [file pone.0130904.s005.docx]

Supplemental Table 1. Primers used in construction of FLAG-tagged CrtO and CrtQ *Synechocystis* strains.

| Primers for cloning/RF cloning | Sequences (5’-3’) |
| --- | --- |
| **CrtO-FLAG** | |
| Cloning of flanking regions and *crtO* gene | forward primer: GGGGGACAGAGGACAAAAGG  reverse primer: TAAACACAGGGCGGCATTGA |
| Insertion of 3xFLAG-tag by RF cloning | dsDNA mega primer: GTCTTTTTAAAACAACAACGTCGTTTTTGGGATTATAAAGATCATGATGGCGATTATAAAGATCATGATATTGATTATAAAGATGATGATGATAAATAATAAAGCAGAAAGCCTAAATCAACATACTAAAAAATG |
| Cm^R^ cassette insertion | forward primer: CGATTATAAAGATCATGATATTGATTATAAAGATGATGATGATAAATAAATGCCATGGAGAGTAAAATCCTCAGTGC  reverse primer: CATTTTTTAGTATGTTGATTTAGGCTTTCTGCTTTACGCCCCGCCCTGCC |
| Segregation screening | forward primer (100bp upstream *crtO* start codon):  GAAAAATTCATTCCAGGCGAT  reverse primer (100bp downstream *crtO* stop codon):  ATATGCTCACCCCAAACGTC |
| **CrtQ-FLAG** | |
| Cloning of flanking regions and *crtQ* gene | forward primer: GGTCAACCTTCACGTCTCCC  reverse primer: TCTAGGGAAGCCGGGGTAAA |
| Insertion of 3xFLAG-tag by RF cloning | dsDNA mega primer:  CAAACTGCAGTCCTGGCATCTCAGGATTATAAAGATCATGATGGCGATTATAAAGATCATGATATTGATTATAAAGATGATGATGATAAATAATAATTTTGGTAAACATCGGCAAAACCCATGG |
| CmR cassette insertion | forward primer:  CATGATGGCGATTATAAAGATCATGATATTGATTATAAAGATGATGATGATAAATAAATGCCATGGAGAGTAAAATCCTCAGTGCCGTC  reverse primer:  CATGGGTTTTGCCGATGTTTACCAAAATTACGCCCCGCCCTGCCACTC |
| Segregation screening | forward primer (100bp upstream *crtQ* start codon):  AACGGGATAGAGAACGGAAC  reverse primer (100bp downstream *crtQ* stop codon):  CGGTCATAAAAACGCACTG |
